# Production of a Thermostable Chitosanase from Shrimp Heads via *Paenibacillus mucilaginosus* TKU032 Conversion and its Application in the Preparation of Bioactive Chitosan Oligosaccharides

**DOI:** 10.3390/md17040217

**Published:** 2019-04-10

**Authors:** Chien Thang Doan, Thi Ngoc Tran, Van Bon Nguyen, Anh Dzung Nguyen, San-Lang Wang

**Affiliations:** 1 Department of Chemistry, Tamkang University, New Taipei City 25137, Taiwan; doanthng@gmail.com (C.T.D.); tranngoctnu@gmail.com (T.N.T.); 2 Department of Science and Technology, Tay Nguyen University, Buon Ma Thuot 630000, Vietnam; bondhtn@gmail.com; 3 Institute of Biotechnology and Environment, Tay Nguyen University, Buon Ma Thuot 630000, Vietnam; nadzungtaynguyenuni@yahoo.com.vn; 4 Life Science Development Center, Tamkang University, New Taipei City 25137, Taiwan

**Keywords:** chitin, chitosan, *Paenibacillus*, chitosanase, chitosan oligomers, α-glucosidase inhibitor, antioxidant

## Abstract

Chitosanase has attracted great attention due to its potential applications in medicine, agriculture, and nutraceuticals. In this study, *P. mucilaginosus* TKU032, a bacterial strain isolated from Taiwanese soil, exhibited the highest chitosanase activity (0.53 U/mL) on medium containing shrimp heads as the sole carbon and nitrogen (C/N) source. Using sodium dodecyl sulfate-polyacrylamide gel electrophoresis (SDS-PAGE) analysis, a chitosanase isolated from *P. mucilaginosus* TKU032 cultured on shrimp head medium was determined at approximately 59 kDa. The characterized chitosanase showed interesting properties with optimal temperature and thermal stability up to 70 °C. Three chitosan oligosaccharide (COS) fractions were isolated from hydrolyzed colloidal chitosan that was catalyzed by TKU032 chitosanase. Of these, fraction I showed the highest α-glucosidase inhibitor (aGI) activity (65.86% at 20 mg/mL); its inhibitory mechanism followed the mixed noncompetitive inhibition model. Fractions II and III exhibited strong 2,2-diphenyl1-picrylhydrazyl (DPPH) radical scavenging activity (79.00% at 12 mg/mL and 73.29% at 16 mg/mL, respectively). In summary, the COS fractions obtained by hydrolyzing colloidal chitosan with TKU032 chitosanase may have potential use in medical or nutraceutical fields due to their aGI and antioxidant activities.

## 1. Introduction

Chitosan is a polymer composed of β-1,4 linked d-glucosamine with varying amounts (under 50%) of *N*-acetyl-d-glucosamine [1]. Chitosan is of interest to many researchers as it exhibits various biological activities and has many biotechnological uses [2,3,4]. Unfortunately, chitosan has poor solubility at neutral pH, potentially limiting its application. Chitosan oligosaccharides (COS) are products obtained from hydrolyzed chitosan with an average MW of less than 3900 Da and degrees of polymerization (DP) under 20 [5]. Unlike chitosan, COS possess great solubility in water. Furthermore, COS also demonstrate antidiabetic [6,7], prebiotic [8,9], antioxidant [5,8], anti-inflammatory [5,10], anticancer [10], antitumor [11], and antibacterial biological activities [12,13]. The enzymatic method was recently reported as an efficient and environmentally friendly process for producing bioactive COS [5,8,9,14,15,16]. However, the high cost of enzyme production limits its application on a larger scale. As such, an inexpensive and efficient protocol for producing chitinolytic enzymes and converting chitosan into bioactive COS is needed.

Chitosanase (EC 3.2.1.132) is a group of enzymes which hydrolyzes chitosan [17]. It is a useful tool for depolymerizing chitosan into oligosaccharides with various biological activities and degrees of DP [5,9]. Important sources for chitosanase production have primarily been found in bacteria, including *Paenibacillus* [9,18], *Bacillus* [5,19,20,21,22,23], *Serratia* [24], and *Streptomyces* [25]. Until now, the common C/N sources for chitosanase production via bacteria were chitin and chitosan. Commercialized chitin and chitosan products are mostly prepared from fish processing by-products, such as shrimp shells, crab shells, or squid pens, using chemical methods like strong alkaline and acid treatments to remove proteins and mineral salts. To reduce costs, shrimp shells, shrimp heads, crab shells and squid pens have been used as the sole C/N sources for enzyme production via microbial conversion [18,19,20,21,22,26,27,28,29,30,31].

Recently, several strains of *Paenibacillus* showed excellent ability in producing α-glucosidase inhibitors [32,33,34,35,36], proteases [18], chitosanase [9,18], and exopolysaccharides [37,38] when marine chitinous materials were used as the sole C/N source. In the previous report, *P. mucilaginosus* TKU032, a bacterial strain isolated from Taiwanese soil, produced exopolysaccharides with high antioxidant activity from a medium containing squid pens [37]. Moreover, the culture supernatant also showed chitinolytic, proteolytic and aGI activities [18]. This suggests that *P. mucilaginosus* TKU032 has the potential to produce various biological activities using low-cost chitinous materials. However, an investigation into TKU032’s enzyme production was not fully explored. 

In this study, chitosanase production of *P. mucilaginosus* TKU032 was tested using four kinds of marine chitinous materials: squid pens powder (SPP), shrimp heads powder (SHP), demineralized shrimp shells powder (deSSP), and demineralized crab shells powder (deCSP) to determine the most suitable C/N source for bioconversion. The isolation and characterization of *P. mucilaginosus* TKU032 chitosanase was performed. Furthermore, the COS obtained by hydrolyzing colloidal chitosan with *P. mucilaginosus* TKU032 chitosanase were fractionated and characterized. The aGI and antioxidant activities of the COS fractions were also estimated and compared to commercial compounds.

## 2. Results and Discussion

### 2.1. Screening of Chitinous Materials as Sole C/N Source for Chitosanase Production

**Figure 1:**
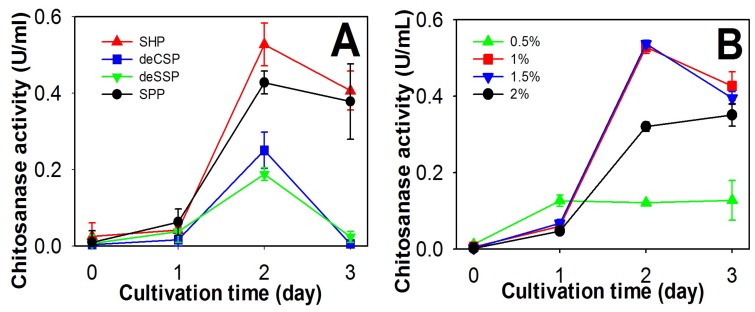
Production of chitosanase by *P. mulaginosus* TKU032; (**A**) using different chitin-containing materials as the C/N source; (**B**) using different concentrations of SHP.

To explore chitosanase production by *P. mucilaginosus* TKU032, four chitinous materials from fish processing (SPP, SHP, deCSP, and deSSP) were used as the sole C/N sources during incubation. As shown in Figure 1, TKU032 chitosanase was found to exhibit the highest activity at day 2 of the cultivation on all types of chitinous material sources, in which the maximum enzyme activity was expressed with SHP (0.53 U/mL) higher than SPP (0.42 U/mL, *p*-value 0.0384), deCSP (0.25 U/mL, *p*-value 0.0028), and deSSP (0.18 U/mL, *p*-value 0.0005), respectively. The reason might be related to the difference in protein/chitin/mineral salts ratio of each chitinous material, in which SHP contents had higher amounts of protein and mineral salts but lower amount of chitin than those of the other materials [36,39]. This result was also unlike previous reports, which suggested that chitosanase from *Paenibacillus* strains often show the highest activity on medium containing SPP [9,18]. 

In the search to find a suitable and cost effective source for producing bioactive compounds via microorganism conversion, shrimp heads, a by-product from shrimp processing, were suggested as potential material as they have been reported as the best sole C/N source for producing chitosanase (*B. cereus* TKU027) [14], aGI (*Staphylococcus* sp. TKU043) [29], nattokinase (*B. subtilis* TKU007) [30], and protease (*B. cereus* TKU022, *B. licheniformis* TKU004) [22,31]. Since TKU032 showed the highest chitosanase activity on medium containing SHP, it was chosen as the sole C/N source.

Further experimentation determined the optimal SHP concentration for chitosanase conversion by *P. mulaginosus* TKU032. As shown in Figure 1B, the highest chitosanase activity was found at 1% SHP (0.53 U/mL) and 1.5% SHP (0.54 U/mL). Since there was no significant difference in maximum chitosanase activity (*p*-value 0.5037) and less material loss, 1% SHP was chosen for subsequent experiments.

### 2.2. Comparison of Chitosanase Production from SHP Using Different Bacteria

Chitinase/chitosanase production by chitinolytic *Paenibacillus* and *Bacillus* strains on SPP and deCSP-containing media were introduced in our previous report [18]. In this study, the potency of SHP in chitinase/chitosanase production via microorganism conversion was also investigated. The results in Table 1 show that all tested species of *Paenibacillus* and *Bacillus* exhibited chitinase and chitosanase activities. The highest chitosanase and chitinase activities were found in *P. mucilaginosus* TKU032 (0.58 U/mL and 0.37 U/mL) and *P. macerans* TKU029 (0.59 U/mL and 0.28 U/mL) with no significant difference in their activities (0.88 and 0.1563 of *p*-value, respectively); the results were better than those of *Paenibacillus* sp. TKU037 (0.05 U/mL and 0.06 U/mL), *Paenibacillus* sp. TKU042 (0.12 U/mL and 0.12 U/mL), *B. subtillis* TKU007 (0.05 U/mL and 0.11 U/mL), and *B. licheniformis* TKU004 (0.01 U/mL and 0.04 U/mL) with a *p*-value range from 0.0004 to 0.0025. By expressing the higher chitinolytic activity on chitosan (0.58 U/mL) than on chitin (0.37 U/mL), *P. mucilaginosus* TKU032 could be considered as a chitosanase-producing bacterium. Similar to the previous study, none of the tested *Paenibacillus* species, including *P. mucilaginosus* TKU032, showed exochitinase activity [18]. Taken together, this result confirmed that SHP was the most suitable C/N source for chitosanase production via *P. mucilaginosus* TKU032 conversion.

### 2.3. Purification and Characterization of Chitosanase

**Figure 2:**
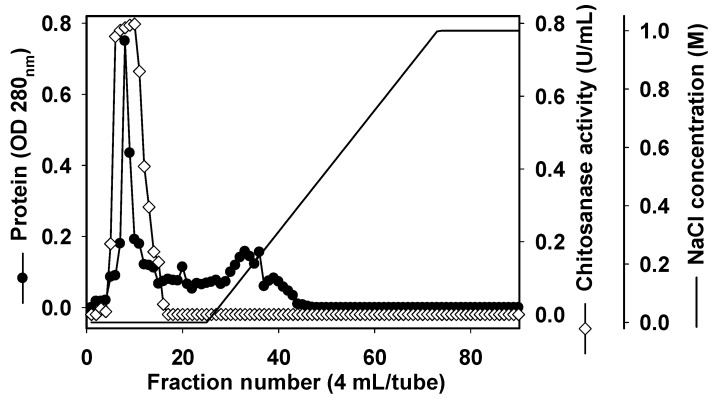
A typical elution profile of chitosanase on Macro-prep High S column.

**Figure 3:**
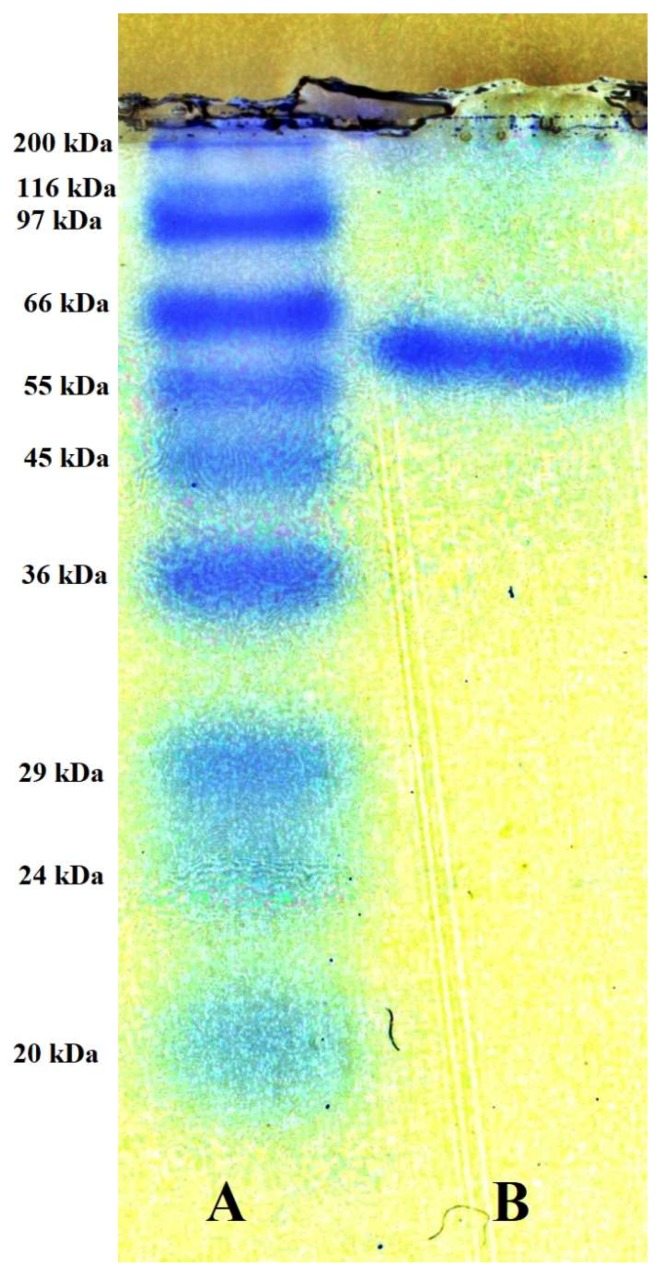
SDS-PAGE analysis of the chitosanase produced by TKU032. A: protein markers; B: Purified chitosanase.

In order to characterize TKU032 chitosanase, a series of steps was used to purify the enzyme, including (NH_4_)_2_SO_4_ precipitation, Macro-prep High S ion exchange chromatography and KW802.5 gel filtration. The chitosanase was eluted by 20 mM Tris-HCl buffer (pH 7) with a linear gradient of 0–1 M NaCl on a Macro-prep High S column. As shown in Figure 2, there was only one peak of chitosanase activity, located at the washing stage of the ion-exchange chromatography separation. This result indicated that pI of TKU032 chitosanase might be ≥7. The peak fractions (fraction numbers 5-15) showing chitosanase activity were collected. After Macro-Prep High S chromatography, 15.98 mg of protein was obtained with an increase in the specific activity from 0.7 U/mg to 4.25 U/mg and a slight reduction in the activity yield from 31.69% to 24.06%. The purification was then confirmed by HPLC gel filtration using the KW802.5 column. Similar to the ion-exchange chromatography, only one peak of chitosanase activity was found by the HPLC analysis (data not showed). After purification, one chitosanase was obtained; its profile is summarized in Table 2. In other reports, only one chitinase/chitosanase was isolated from the culture medium of *Paenibacillus* species [1,9,18,40,41,42,43,44,45,46,47,48]. The specific activity and recovery yield of the purified chitosanase were 5.13 U/mg and 10.94%, respectively. By using SDS-PAGE analysis, the molecular weight (MW) of the TKU032 chitosanase was determined as approximately 59 kDa (Figure 3), which fell within the MW’s range (35–85 kDa) of chitinase/chitosanase from most *Paenibacillus* strains [1,9,18,40,41,42,43,44,45,46,47,48], with an exception from *Paenibacillus* sp. FPU-7 chitinase (150 kDa) [49].

### 2.4. Effects of pH and Temperature on Activity and Stability of Chitosanase

**Figure 4:**
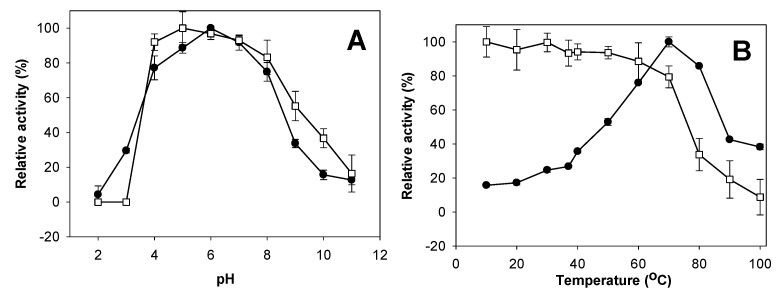
Effect of pH (**A**) and temperature (**B**) on activity (●) and stability (□) of TKU032 chitosanase.

The effect of pH on *P. mucilaginosus* TKU032 chitosanase is shown in Figure 4A. The optimum pH for the enzyme was 6.0. This result is consistent with most research, which showed chitinase/chitosanase from *Paenibacillus* species expressing the highest activity in acidic conditions [1,41,45,46,47], with the exceptions of *P. macerans* TKU029 [9], *P. pasadenensis* NCIM5434 [43] and *P. elgii* HOA73 [42]. *P. mucilaginusus* TKU032 was stable over a broad pH range (Figure 4A), with over 80% of activity retained from pH 4 to 8. Once the pH dropped below 4, activity disappeared completely, whereas 50% of activity was retained at pH 9. Several chitinase/chitosanase from *Paenibacillus* showed the same broad pH stability as *P. mucilaginosus* TKU032 [9,46,48,50]. 

The effect of temperature on the activity and stability of *P. mucilaginosus* TKU032 chitosanase were studied herein (Figure 4B). The optimal temperature was 70 °C, but even at 80 °C, it still showed more than 80% activity. *P. mucilaginosus* TKU032 chitosanase demonstrated thermal stability up to 70 °C, with over 80% of activity retained. This suggests that both the optimal temperature and thermal stability of *P. mucilaginosus* TKU032 chitosanase were higher than most chitinase/chitosanase from other *Paenibacillus* species [9,41,42,43,44,45,46,47]; only a chitosanase from *Paenibacillus* sp. 1794 showed similar results [1]. Due to its higher thermal stability, *P. mucilaginosus* TKU032 chitosanase may have potential use in industrial applications.

### 2.5. Effect of Metal Ions on Activity of Chitosanase

The effects of known divalent metals and enzyme inhibitors on the activity of TKU032 chitosanase were examined; the results are summarized in Table 3. All chemicals reduced the activity of TKU032 chitosanase, a result markedly different from other reports, which showed that the addition of some metal ions, Na^+^ and Fe^2+^ for instance, could enhance the activity of chitosanases from *Paenibacillus* [9,50].

### 2.6. Substrate Specificity of Chitosanase

**Figure 5:**
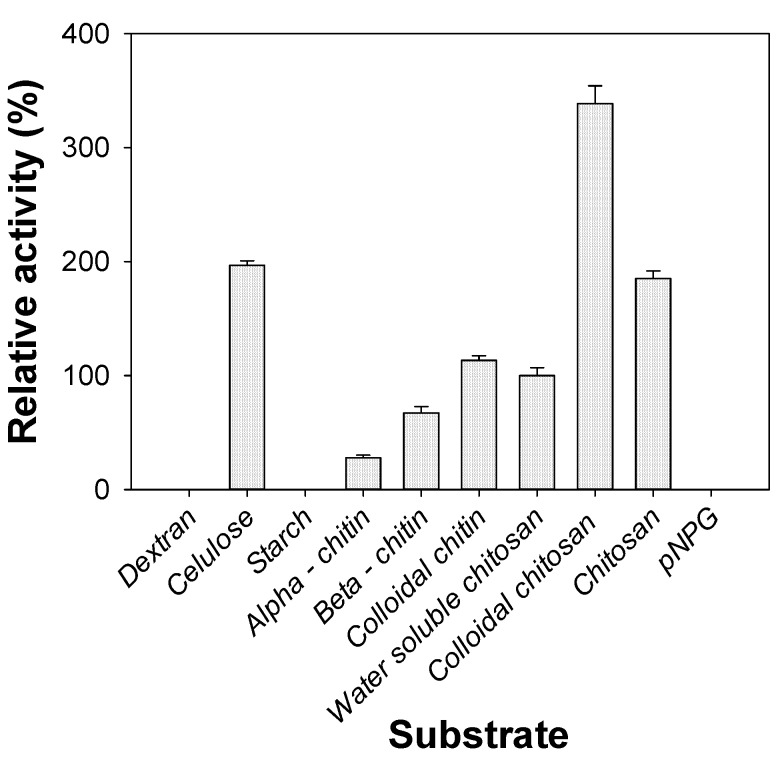
Substrate specificity of *P. mucilaginosus* TKU032 chitosanase.

The activity of the purified *P. mucilaginosus* TKU032 chitosanase on various substrates is shown in Figure 5. Chitosanase activity was expressed in the descending order of colloidal chitosan > cellulose > chitosan > colloidal chitin > water-soluble chitosan > β-chitin > α-chitin. No activity was shown on dextran, starch, or *p*-nitrophenyl-*N*-acetyl-β-d-glucosaminide (*p*NPG). These results suggest that the physical form of the substrate had a strong effect on the rate of hydrolysis. Unlike *P. macerans* TKU029 chitosanase, where the most suitable substrate was water-soluble chitosan [9], *P. mucilaginosus* TKU032 chitosanase demonstrated excellent activity on colloidal chitosan (338.74%), followed by chitosan powder (185.38%). It also showed good activity on chitin substrates, expressing 113.24%, 67.19%, and 27.67% on colloidal chitin, β-chitin, and α-chitin, respectively, as well as on cellulose (196.84%). These results indicate that *P. mucilaginosus* TKU032 chitosanase could express good activity on various types of substrates, including cellulose, chitin, and chitosan. Since *p*NPG is a specific substrate of exochitinase, it was logical that *P. mucilaginosus* TKU032 chitosanase would not show any exochitinase activity.

### 2.7. COS Production

**Figure 6:**
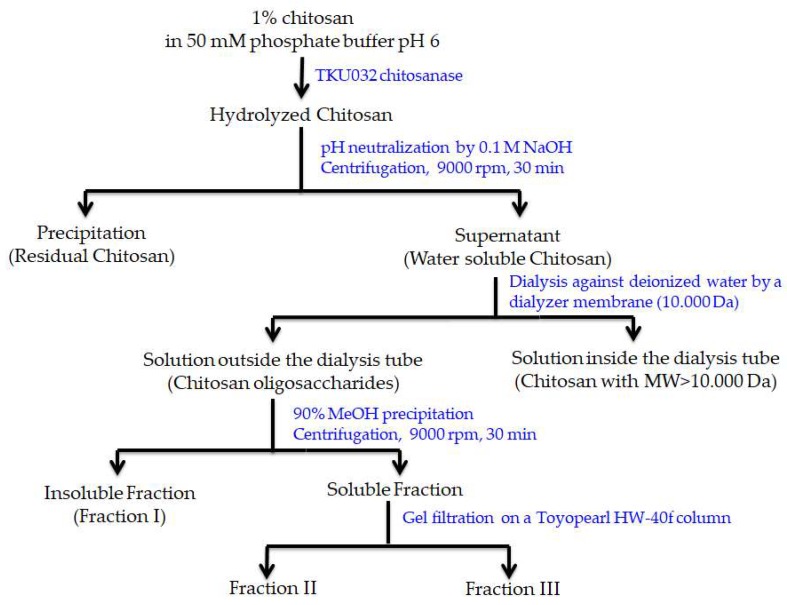
Flow chart for the isolation of COS produced by hydrolyzing chitosan with TKU032 chitosanase

**Figure 7:**
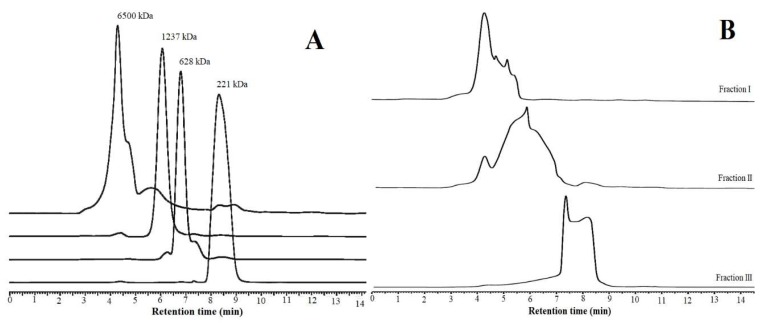
HPLC profiles of chitosan oligosaccharide fractions: (**A**) references; (**B**) chitosan oligosaccharide fractions

Since *P. mucilaginosus* TKU032 chitosanase expressed the best activity on colloidal chitosan, this substrate was used to produce COS using the enzyme hydrolysis method. After the reaction finished, a series of steps were used to separate COS from the hydrolyzed solution, including pH neutralization, centrifugation, and dialysis by a 10,000 Da membrane. The obtained COS was then fractionated by MeOH 90% precipitation and gel filtration on a Toyopearl HW-40f column. As shown in Figure 6, 3 COS fractions were collected from the hydrolyzed chitosan solution. The molecular weights of the obtained fractions were determined by HPLC analysis using a KS-802 column (Figure 7). The molecular weights of fractions I, II, and III were approximately 1600-6500 Da, 424-6500 Da, and 221-424 Da, respectively. Fraction I only contained COS with higher MW, while fraction II was a mixture of COS with high and low MW, and fraction III included COS with the lowest MW. Compared to the reference peaks, fraction III mainly contained COS with DP from 1 to 2.

### 2.8. Evaluation of Antioxidant and aGI Activities of COS Fractions

**Figure 8:**
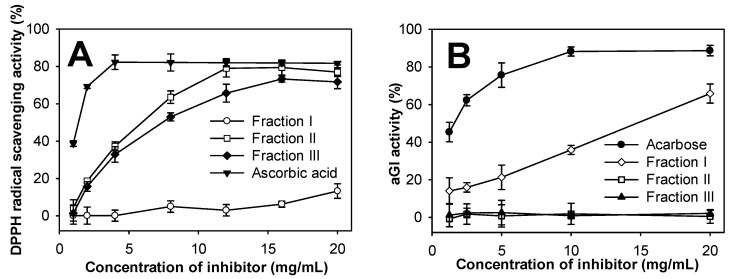
(**A**) antioxidant and (**B**) aGI activities of COS fractions.

Free radicals negatively affect living organisms, resulting in DNA mutation, lipid and protein damage, cancer, and cardiovascular or neurodegenerative diseases [51]. The harmful actions of free radicals could be reduced or prevented by antioxidant compounds. In the current study, the DPPH radical scavenging ability was assayed to explore the antioxidant activity of the three obtained COS fractions. As shown in Figure 8A, within a concentration range of 1–20 mg/mL, only fractions II and III showed DPPH radical scavenging activity with a dose-dependent increase. The maximum antioxidant activity of fraction II was 79.00% at a concentration of 12 mg/mL, while fraction III was 73.29% at 16 mg/mL. This suggests that fraction II is the strongest antioxidant among the three fractions. It is well known that COS is a potential candidate for antioxidant activity; however, its antioxidant ability was strongly affected by its MW [5,8]. The obtained results indicated that COS with lower MW could possess higher antioxidant activity. Moreover, this study also showed that low MW COS with DP > 2 expressed higher DPPH radical scavenging activity than COS with DP ≤ 2. Compared to ascorbic acid, a commercial antioxidant compound, fraction II expressed weaker activity. The two compounds generated maximum activity at concentrations of 4 mg/mL and 12 mg/mL, respectively. However, as there was no significant difference (*p*-value 0.404) between the maximum activity of ascorbic acid (82.29%) and fraction II (79.00%), fraction II may have comparable antioxidant activity. As such, COS fraction II could be an acceptable antioxidant compound for use in the medical or food industries.

**Figure 9:**
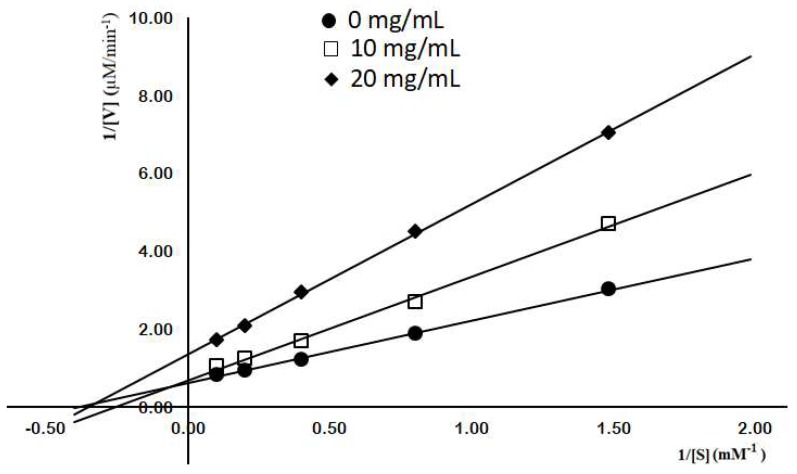
Lineweaver-Burk plot analysis of aGI activity by COS fraction I.

As α-glucosidase inhibitors are an important therapy for type 2 diabetes, the COS fractions were also investigated for α-glucosidase inhibitor activity. As shown in Figure 8B, only fraction I expressed dose-dependent aGI activity within a range of 1–20 mg/mL. At 20 mg/mL, fraction I exhibited 65.86% inhibitory activity on yeast α-glucosidase, which was weaker than acarbose (88.21% at 10 mg/mL) with 0.0005 of *p*-value. Only COS with higher MW showed aGI activity, unlike the results of Jo et al. (2013) who found similar aGI activity in rat α-glucosidase from 3 different molecular weight COS fractions [6]. For further investigation, the inhibition model of fraction I on yeast α-glucosidase was analyzed using a Lineweaver-Burk plot. Figure 9 shows that when the concentration of COS fraction I increased from 0 to 20 mg/mL, 1/V_M_ increased but -1/K_M_ varied. This suggests that the aGI activity of COS fraction I follows the mixed noncompetitive inhibition model. Although chitosan and COS have been widely studied for treating diabetes [52], there have been few reports on their inhibition of α-glucosidase, a key enzyme in the digestive system that hydrolyzes dietary carbohydrates to increase blood glucose concentration. As such, this result could prove a novel contribution to aGI research. 

## 3. Materials and Methods 

### 3.1. Materials

Shrimp shells, squid pens, and crab shells were purchased from Shin-Ma Frozen Food Co. (I-Lan, Taiwan). Shrimp heads was bought from Fwu-Sow Industry (Taichun, Taiwan). Shrimp shells and crab shells were demineralized by acid treatment [8]. All bacterial strains were provided by the Microorganisms and Biochemistry Laboratory, Department of Chemistry, Tamkang University, New Taipei, Taiwan. Chitosan, DPPH, yeast α-glucosidase and 3,5-dinitrosalicylic acid (DNS) reagents were all bought from Sigma-Aldrich Corp. (Singapore). Macro-Prep High S was bought from Bio-Rad (Hercules, CA, USA). All other reagents used were the highest grade available.

### 3.2. Measurement of Chitosanase Activity

The chitosanase activity assay was modified from previously described methods [18]. The substrate solution was prepared by adding water-soluble chitosan into 20 mM Tris-HCl buffer at pH 7 to reach a final concentration of 1% (w/v). The hydrolysis reaction of chitosan was performed with 100 µL of the sample and 100 µ1 of substrate, and maintained at 37 °C in an incubator for 30 min. The DNS method was used to detect the amount of reducing sugar produced, with d-glucosamine used as the reference. The definition of a unit of chitosanase activity was the amount of enzyme catalyzed to produce 1 µmol of reducing sugar in one min. 

### 3.3. Screening of Chitinous Materials as Sole C/N Source for Chitosanase Activity

Four fishery by-products: deCSP, SPP, SHP, and deSSP, were added to 100 mL of basal medium (0.1% K_2_HPO_4_ and 0.05% MgSO4·7H_2_O) in 250 mL Erlenmeyer flasks at similar concentrations (1%) [9]. Fermentation was performed with 1% of seed culture at 37 °C and a shaking speed of 150 rpm for 3 d. The culture supernatant was measured daily for chitosanase activity.

### 3.4. Purification of Chitosanase

Protein precipitation was performed by adding 480 g of (NH_4_)_2_SO_4_ to 600 mL of culture supernatant. The solution was kept at 4 °C overnight. The precipitate was centrifuged at 12,000 x g for 30 min and then dissolved in 50 mL of 20 mM Tris-HCl buffer (pH 7) to produce the crude enzyme solution. The enzyme solution was then loaded onto a Macro-Prep High S column, which had been equilibrated with 20 mM Tris-HCl buffer, and eluted by a linear gradient of 0-1 M NaCl in the same buffer. The obtained fraction, which showed the chitosanase, was then further purified by HPLC analysis using the KW802.5 column following the conditions: mobile phase, 0.3 M NaCl in 20 mM Tris-HCl buffer (pH 7); injection volume, 20 µL; temperature, 20 °C; flowrate, 1mL/min; and detector, ultraviolet 230 nm. The MW of the purified chitosanase was determined using the SDS-PAGE method.

### 3.5. Effects of pH and Temperature on Activity and Stability of Chitosanase

The optimal temperature for the reaction of TKU032 chitosanase was determined over a range of 4–100 °C with 30 min incubation. Thermal stability was determined by treating the enzyme at a range of temperatures for 30 min. The residual activity of the treated enzyme was measured under the standard conditions of the chitosanase activity assay.

The effect of pH on TKU32 chitosanase activity was investigated using the buffer systems, including glycine-HCl, acetate, sodium phosphate, and Na_2_CO_3_-NaHCO_3_. The optimal pH of TKU032 chitosanase was tested using a range of 2 to 11. To investigate the pH stability of TKU032 chitosanase, the enzyme was pre-treated from pH 2 to 11 for 30 min; its residual activity was measured at pH 7, as described above.

### 3.6. Effect of Metal Ions on Chitosanase Activity

TKU032 chitosanase was pre-incubated for 30 min at 37 °C with various metal ion salts and an enzyme inhibitor (5 mM), including: Cu^2+^, Zn^2+^, Mg^2+^, Na^+^, Ba^2+^, Ca^2+^, Fe^2+^, and EDTA. The residual chitosanase activity was then measured, using the methods described above.

### 3.7. Substrate Specificity of Chitosanase

TKU032 chitosanase was incubated in 20 mM Tris-HCl buffer with numerous substrates at 70 °C for 30 min. These included: dextran, potato starch, cellulose powder, α-chitin powder, β-chitin powder, chitosan powder, colloidal chitin, colloidal chitosan, and *p*NPG. The enzyme activity in water-soluble chitosan was used as a control.

### 3.8. Antioxidant Activity Assay

DPPH radical scavenging activity was tested as per the previous methods, with modifications [51]. Briefly, 20 µL of sample was mixed with 980 µL DPPH-methanol solution and the mixture was kept in the dark for 20 min. The solution was then measured for optical density at 517 nm. The antioxidant activity was calculated using formula (1):

DPPH radical scavenging activity (%) = (A_1_ − A_2_)/A_1_ × 100 (1)

where A is the optical density of a blank sample and B is the optical density of the sample solution.

### 3.9. aGI Activity Assay

The aGI activity test followed the previously described methods [18]. In brief, 10 µL of sample was mixed with an equal amount of yeast α-glucosidase solution (1 U) and 100 µL phosphate buffer (100 mM, pH 6.8). The mixture was immediately incubated at 37 °C for 30 min. The reaction of the enzyme and substrate was started by adding 10 µL *p*-nitrophenyl glucopyranoside to the mixture, and then incubating at 37 °C for a further 30 min. 130 µL Na_2_CO_3_ solution (1 M) was added to the mixture to stop the reaction. The final solution was measured at 410 nm. The aGI activity was calculated using the following formula (2):

aGI activity (%) = (B_1_ − B_2_)/B_1_ × 100 (2)

where B_1_ is the optical density of the blank sample, and B_2_ is the optical density of the sample solution.

## 4. Conclusions

Chitosan oligosaccharides with various degrees of polymerization and biological activities could be efficiently produced using chitosanase to hydrolyze chitosan. In the current study, *P. mucilaginosus* TKU032 achieved the highest chitosanase production using shrimp head powder, an inexpensive material, as the sole C/N source. The *P. mucilaginosus* TKU032 chitosanase was purified and unlike other *Paenibacillus* strains, had a MW of 59 kDa and a high optimal temperature (70 °C). In order to evaluate the bioactivity of COS, the oligomers obtained by hydrolyzing colloidal chitosan with TKU032 chitosanase were fractionalized and tested for aGI and antioxidant activities. The COS fraction with the highest MW (fraction I) demonstrated aGI activity, whereas the COS fractions with lower MW (fractions II and III) showed antioxidant activity. For the first time, the inhibitory mechanism of COS on yeast α-glucosidase was investigated; the results suggest that it follows the mixed noncompetitive inhibition model. As such, chitosanase from *P. mucilaginosus* TKU032 may have potential applications in bioactive COS production for the food and pharmaceutical industries.

## Figures and Tables

**Table 1:** Comparison of chitosanase, chitinase, and exochitinase production by different *Paenibacillus* and *Bacillus* strains.

Bacterial Strain	Chitosanase Activity (U/mL)	Chitinase Activity (U/mL)	Exochitinase Activity (U/mL)
*P. mucilaginosus* TKU032	0.58 ± 0.10	0.37 ± 0.04	-
*P. macerans* TKU029	0.59 ± 0.04	0.28 ± 0.08	-
*Paenibacillus* sp. TKU037	0.05 ± 0.02	0.06 ± 0.05	-
*Paenibacillus* sp. TKU042	0.12 ± 0.04	0.12 ± 0.05	-
*Bacillus licheniformis* TKU004	0.01 ± 0.01	0.04 ± 0.01	10.21 ± 0.89
*B. subtillis* TKU007	0.05 ± 0.02	0.11 ± 0.01	-

Bacterial strains were cultured in 100 mL of liquid medium in an Erlenmeyer flask (250 mL) containing 1% SHP, 0.1% K_2_HPO_4_ and 0.05% MgSO_4_·7H_2_O in a shaking incubator for 2 d at 37 °C.

**Table 2:** Purification of chitosanase from *P. mucilaginosus* TKU032.

Step	Total Protein (mg)	Total Activity (U)	Specific Activity (U/mg)	Recovery (%)	Purification (Fold)
Cultural supernatant	1499.13	282.28	0.19	100.00	1.00
(NH_4_)_2_SO_4_ precipitation	126.97	89.44	0.70	31.69	3.74
Macro-Prep High S	15.98	67.92	4.25	24.06	22.57
KW-802.5	5.13	30.89	6.03	10.94	32.01

*P. mucilaginosus* TKU032 was cultured in 100 mL of liquid medium in an Erlenmeyer flask (250 mL) containing 1% SHP, 0.1% K_2_HPO_4_ and 0.05% MgSO_4_·7H_2_O in a shaking incubator for 2 d at 37 °C.

**Table 3:** Effect of metal ions on the activity of chitosanase

Metal Ion	Relative Activity (%)
Control	100.00 ± 6.39
Cu^2+^	74.37 ± 3.95
Zn^2+^	76.78 ± 3.25
Mg^2+^	84.39 ± 4.51
Na^+^	91.71 ± 5.21
Ba^2+^	62.14 ± 12.29
Ca^2+^	77.07 ± 5.68
Fe^2+^	65.13 ± 6.77
EDTA	84.39 ± 7.53
